# NTB-A and 2B4 Natural Killer Cell Receptors Modulate the Capacity of a Cocktail of Non-Neutralizing Antibodies and a Small CD4-Mimetic to Eliminate HIV-1-Infected Cells by Antibody-Dependent Cellular Cytotoxicity

**DOI:** 10.3390/v16071167

**Published:** 2024-07-20

**Authors:** Lorie Marchitto, Alexandra Tauzin, Mehdi Benlarbi, Guillaume Beaudoin-Bussières, Katrina Dionne, Étienne Bélanger, Debashree Chatterjee, Catherine Bourassa, Halima Medjahed, Derek Yang, Ta-Jung Chiu, Hung-Ching Chen, Amos B. Smith III, Jonathan Richard, Andrés Finzi

**Affiliations:** 1 Centre de Recherche du CHUM, Montreal, QC H2X 0A9, Canadajonathan.richard.1@umontreal.ca (J.R.); 2 Département de Microbiologie, Infectiologie et Immunologie, Université de Montréal, Montreal, QC H2X 0A9, Canada; 3 Department of Chemistry, School of Arts and Sciences, University of Pennsylvania, Philadelphia, PA 19104, USA

**Keywords:** NK cell activating receptors, ADCC, CD4 mimetics, nnAbs, plasma from PLWH

## Abstract

Natural Killer (NK) cells have the potential to eliminate HIV-1-infected cells by antibody-dependent cellular cytotoxicity (ADCC). NK cell activation is tightly regulated by the engagement of its inhibitory and activating receptors. The activating receptor CD16 drives ADCC upon binding to the Fc portion of antibodies; NK cell activation is further sustained by the co-engagement of activating receptors NTB-A and 2B4. During HIV-1 infection, Nef and Vpu accessory proteins contribute to ADCC escape by downregulating the ligands of NTB-A and 2B4. HIV-1 also evades ADCC by keeping its envelope glycoproteins (Env) in a “closed” conformation which effectively masks epitopes recognized by non-neutralizing antibodies (nnAbs) which are abundant in the plasma of people living with HIV. To achieve this, the virus uses its accessory proteins Nef and Vpu to downregulate the CD4 receptor, which otherwise interacts with Env and exposes the epitopes recognized by nnAbs. Small CD4-mimetic compounds (CD4mc) have the capacity to expose these epitopes, thus sensitizing infected cells to ADCC. Given the central role of NK cell co-activating receptors NTB-A and 2B4 in Fc-effector functions, we studied their contribution to CD4mc-mediated ADCC. Despite the fact that their ligands are partially downregulated by HIV-1, we found that both co-activating receptors significantly contribute to CD4mc sensitization of HIV-1-infected cells to ADCC.

## 1. Introduction

Increasing evidence highlights the central role of Fc-mediated antibody effector functions in eliminating HIV-1-infected cells in vitro and in vivo [1,2]. Among these Fc effector functions, antibody-dependent cellular cytotoxicity (ADCC) mediated by Naturel Killer (NK) cells is a major mechanism to clear HIV-1-infected cells [3,4,5,6,7,8,9]. NK cells express a complex array of inhibitory and activating receptors that tightly control their effector function including ADCC. Among NK cell activating receptors, CD16 (FcγRIIIa) has increased affinity for IgG Fc fragment [10]. CD16 engagement by antibodies triggers the release of NK cells’ lytic granules, leading to target cell death [11].

Non-neutralizing antibodies (nnAbs) targeting the HIV envelope glycoprotein (Env) are readily elicited after infection and mediate potent ADCC when their epitopes are exposed in downstream “open” Env conformations [12,13,14,15,16]. Viral accessory proteins Nef and Vpu tightly regulate Env conformation by downregulating cell surface CD4 [17,18,19,20], preventing a premature encounter between Env and CD4 which would otherwise result in the exposure of these vulnerable epitopes [14,15,16,21,22,23,24,25,26]. Thus, primary viruses sample a “closed” conformation [27] where the epitopes recognized by these nnAbs are occluded. This mechanism contributes to the resistance of HIV-1-infected cells to ADCC [14,15,26,28,29]. Env accumulation at the cell surface is also indirectly regulated by Vpu-mediated counteraction of the host restriction factor Tetherin/BST-2 [30,31,32,33]. Tetherin restricts the budding of viral particles resulting in Env accumulation at the cell surface [14,30,31,32]. In addition, HIV-1 accessory proteins Nef and Vpu interfere with NK cells by downregulating ligands of the activating and co-activating NK cell receptors from the surface of HIV-1-infected cells [34,35,36,37,38,39].

Despite the significant role of NK cells in eliminating HIV-1-infected cells, their capacity to effectively clear and control viremia remains limited due to the difficulty of antibodies to recognize Env at the surface of infected cells. One strategy to overcome this limitation consists of forcing Env to sample “open” conformations using small CD4-mimetic compound (CD4mc). CD4mc, including CJF-III-288, bind within the Env Phe43 cavity and trigger conformational changes similar to those induced by membrane-bound CD4, resulting in the exposure of epitopes recognized by nnAbs [3,16,23,24,40,41]. This strategy has proven effective in vitro and in vivo using CD4mc and plasma from people living with HIV (PLWH) or cocktails of nnAbs [3,16,23,24,40,42]. Recently, we described a cocktail of nnAbs combined with a new indoline CD4mc, CJF-III-288, that mediated potent ADCC against a panel of primary viruses [43]. The nnAb part of the cocktail is comprised of anti-cluster A Ab, anti-coreceptor binding site (CoRBS) Ab, and anti-gp41 cluster I Ab at equimolar ratios [43].

While CD16 engagement induces NK cell degranulation, their lytic activity is maintained and increased through synergistic interactions with other NK cell activating receptors, such as DNAM-1, NKG2D, and co-activating receptors, including NTB-A and 2B4 [36,39,44,45,46,47]. NTB-A and 2B4 are part of the Signaling Lymphocyte Activation Molecules (SLAM) family [48]. NTB-A makes homophilic interaction and is expressed both at the surface of human NK cells and CD4 T cells [49]. In contrast, 2B4 is expressed at the surface of NK cells and binds CD48, a cell surface protein expressed on CD4 T cells [50,51]. Both co-activating receptors NTB-A and 2B4 play a central role in ADCC response by their synergistic activity with CD16, NKG2D, and DNAM-1 [36,39]. Interestingly, their respective ligands (NTB-A and CD48) were shown to be downmodulated at the surface of primary CD4 T cells infected in vitro [36,37,39] as well as ex vivo-expanded CD4 T cells isolated from PLWH [52]. This downmodulation mediated by Vpu was shown to contribute to reduce the ADCC directed against HIV-1-infected cells [36,39]. However, while their ligands are incompletely downregulated by Vpu, their remaining expression on HIV-1-infected cells might be sufficient for NTB-A and 2B4 to synergize with CD16, thereby still impacting ADCC. Furthermore, NTB-A and 2B4 expression on NK cells from PLWH was suggested to remain stable over time, as opposed to the activating receptors NKp30, NKp44, NKp46, and NKG2D [53,54,55]. Given the efficacy of a recently developed cocktail to eliminate HIV-1-infected cells by ADCC and the central role of NTB-A and 2B4 in synergizing with CD16, we wanted to characterize the role of these co-activating receptors in the elimination of HIV-1-infected cells by nnAbs combined with CD4mc.

## 2. Materials and Methods

### 2.1. Ethics Statement

Written informed consent was obtained from all study participants, and the research adhered to the ethical guidelines of CRCHUM and was reviewed and approved on 21 September 2023 by the CRCHUM Institutional Review Board (Ethics Committee approval number MP-02-2024-11734). The research adhered to the standards indicated by the Declaration of Helsinki.

### 2.2. Cell Lines and Primary Cells

HEK293T human embryonic kidney cells (obtained from ATCC) were maintained at 37 °C under 5% CO_2_ in Dulbecco’s Modified Eagle Medium (DMEM) (Wisent, Saint-Jean-Baptiste, QC, Canada), supplemented with 5% fetal bovine serum (FBS) (VWR, Atlanta, GA, USA) and 100 U/mL penicillin/streptomycin (Wisent). Primary human PBMCs and CD4^+^ T cells were isolated, activated, and cultured as previously described [15]. Briefly, PBMCs were obtained by leukapheresis from four HIV-negative individuals (three males and one female) and primary CD4^+^ T lymphocytes were purified from resting PBMCs with negative selection using immunomagnetic beads per the manufacturer’s instructions (StemCell Technologies, Vancouver, Canada) and were activated with phytohemagglutinin-L (10 µg/mL) for 48 h. CD4 T cells were maintained in RPMI 1640 complete medium supplemented with rIL-2 (100 U/mL).

### 2.3. Plasmids, Antibodies, and Plasma

The following antibodies were used to assess cell-surface Env staining and ADCC responses: A32 (plasmids for HC and LC were kindly provided by James Robinson); anti-CoRBS 17b (plasmids for HC and LC were kindly provided by James Robinson); anti-gp41 nnAb 246D (plasmids for HC, Cat#13741 and LC, Cat#13742 were provided by NIH AIDS Reagent Program [56]), Human NTB-A/SLAMF6 APC-conjugated (R&D System, Minneapolis, MN, USA), and APC or PerCp/Cy5.5 anti-human CD48 (clone BJ40, Biolegend) were used to assess respective cell surface protein levels. Goat anti-human and anti-mouse antibodies pre-coupled to Alexa Fluor 647 (Invitrogen, Waltham, MA, USA) were used as secondary antibodies in flow cytometry experiments when required. Plasma from PLWH were collected, heat-inactivated and conserved at −80 °C until use. Characteristics of the PLWH donors are described in Table 1. Mouse anti-human CD4 (clone OKT4, FITC-conjugated; Biolegend, San Diego, CA, USA) and anti-p24 mAb (clone KC57; PE-conjugated;Beckman Coulter, Brea, CA, USA) or mouse anti-human CD4 (clone OKT4, PE-conjugated; Biolegend, San Diego, CA, USA) and anti-p24 mAb (clone KC57; FITC-conjugated; Beckman Coulter) were used to identify the productively infected cells as previously described [26].

### 2.4. Antibody Expression and Purification

FreeStyle 293F cells (Thermo Fisher Scientific, Waltham, MA, USA) were grown in FreeStyle 293F medium (Thermo Fisher Scientific) to a density of 1 × 106 cells/mL at 37 °C with 8% CO_2_ with regular agitation (150 rpm). Cells were transfected with plasmids expressing the light and heavy chains of A32, 17b, and 246D Abs using ExpiFectamine 293 transfection reagent, as directed by the manufacturer (Thermo Fisher Scientific). One week later, the cells were pelleted and discarded. The supernatants were filtered (0.22 μm pore size filter), and antibodies were purified by protein A affinity columns, as directed by the manufacturer (Cytiva, Marlborough, MA, USA). Antibodies were dialyzed against phosphate-buffered saline (PBS) and stored in aliquots at −80 °C. To assess purity, recombinant proteins were loaded on SDS-PAGE polyacrylamide gels in the presence or absence of β-mercaptoethanol and stained with Coomassie blue.

### 2.5. Small Molecules

The small-molecule CD4-mimetic compound (CD4mc) CJF-III-288 was synthesized as described previously [57]. The compound was dissolved in dimethyl sulfoxide (DMSO) at a stock concentration of 10 mM and diluted at 50 µM concentration in phosphate-buffered saline (PBS) for cell surface staining or in RPMI-1640 complete medium for ADCC assays.

### 2.6. Proviral Constructs

The vesicular stomatitis virus G (VSV-G)-encoding plasmid was previously described [58]. Transmitted/Founder (T/F) infectious molecular clone (IMC) CH058 (CH058TF) was previously described [59,60].

### 2.7. Viral Production and Infection

Vesicular stomatitis virus G (VSV-G)-pseudotyped HIV-1 viruses were produced by co-transfection of HEK293T cells with an HIV-1 proviral construct and a VSV-G-encoding vector using the PEI reagent (Polysciences). Two days post-transfection, cell supernatants were harvested, clarified by low-speed centrifugation (300× *g* for 5 min), and concentrated by ultracentrifugation at 4 °C (100,605× *g* for 1 h) over a 20% sucrose cushion. Pellets were resuspended in fresh RPMI, and aliquots were stored at −80 °C until use. To achieve a similar level of infection in primary CD4^+^ T cells, VSV-G-pseudotyped HIV-1 viruses were produced and titrated as previously described [15,16]. Viruses were then used to infect activated primary CD4^+^ T cells from HIV-1-negative donors by spin infection at 800× *g* for 1 h in 96-well plates at 25 °C. All experiments using VSV-G-pseudotyped HIV-1 isolates were conducted in a biosafety level 3 laboratory following manipulation protocols accepted by the CRCHUM Biosafety Committee, which respects the requirements of the Public Health Agency of Canada.

### 2.8. Flow Cytometry Analysis of Cell Surface and Intracellular Staining

Cell surface staining of infected primary CD4^+^ T cells was performed 48 h post-infection, as previously described [16,40]. Infected CD4 T cells were incubated for 30 min at 37 °C with anti-Env mAbs (5 µg/mL) or with plasma (dilution 1:1000). Cells were then washed once with PBS and stained with the anti-human Alexa Fluor 647-conjugated secondary antibody (2 μg/mL), AquaVivid (dilution 1:1000), and anti-CD4 FITC (1:1000) for 20 min at room temperature. Alternatively, primary CD4 T cells were stained with APC-conjugated anti-NTB-A (dilution 1:100) or anti-CD48 (dilution 1:100) for 30 min at 37 °C. After one more PBS wash, cells were fixed in a 2% PBS-formaldehyde solution. Infected cells were then permeabilized using the Cytofix/Cytoperm Fixation/Permeabilization Kit (BD Biosciences, Mississauga, ON, Canada) and stained intracellularly using PE-conjugated mouse anti-p24 mAb (clone KC57; Beckman Coulter, Brea, CA, USA; 1:100 dilution) or FITC-conjugated mouse anti-p24 mAb (clone KC57; Beckman Coulter, Brea, CA, USA; 1:100 dilution). The gating strategy is depicted in Supplemental Figure S3A.

### 2.9. FACS-Based ADCC Assay

Measurement of ADCC using a FACS-based assay was performed at 48 h post-infection as previously described [16]. Of note, this assay specifically measures the killing of productively infected cells (CD4^low^p24^+^) and is not affected by the presence of uninfected bystander cells [26,61]. Briefly, infected purified primary CD4^+^ T cells were stained with viability dye (AquaVivid; ThermoFisher Scientific, Waltham, MA, USA) and cell proliferation dye (eFluor670; eBioscience, San Diego, CA, USA) and used as target cells. Autologous PBMC effector cells, stained with another cellular marker (cell proliferation dye eFluor450; eBioscience) were added as an effector: target ratio of 10:1 in 96-well V-bottom plates (Corning, Corning, NY, USA). nnAbs A32/17b/246D mAbs (5 µg/mL total) or plasma (1:1000) were added to appropriate wells and cells were incubated for 15 min at room temperature. The plates were subsequently centrifuged for 1 min at 300× *g* and incubated at 37 °C, 5% CO_2_ for 5–6 h before being fixed in a 2% PBS-formaldehyde solution. Infected cells were identified by intracellular staining for HIV-1 p24 protein and by detection of cell surface CD4, as described above. Alternatively, effector cells were preincubated for 30 min in the presence of anti-NTB-A and/or anti-2B4 antibodies or their matched IgG isotype control (10 µg/mL) prior to being directly incubated with target cells in the absence or presence of A32/17b/246D mAbs or plasma for blockade experiments. Samples were acquired on a Fortessa cytometer (BD Biosciences, Mississauga, Ontario, Canada), and data analysis was performed using FlowJo v10.5.3 (Tree Star, Ashland, OR, USA). The gating strategy is depicted in Supplemental Figure S3B.

The percentage of ADCC was calculated with the following formula: [(% of CD4^low^p24^+^ cells in Targets plus Effectors) − (% of CD4^low^p24^+^ cells in Targets plus Effectors in presence of plasma or mAbs)/(% of CD4^low^p24^+^ cells in Targets) × 100] by gating on productively infected live target cells.

### 2.10. Statistical Analysis

Statistics were analyzed using GraphPad Prism version 10 (GraphPad). Every data set was tested for statistical normality and this information was used to apply the appropriate (parametric or nonparametric) statistical test. *p* values < 0.05 were considered significant; significance values are indicated as * *p* < 0.05, ** *p* < 0.01, *** *p* < 0.001, **** *p* < 0.0001.

## 3. Results

### 3.1. CD4mc Enables nnAbs to Recognize and Eliminate HIV-1-Infected Cells by ADCC

**Figure 1:**
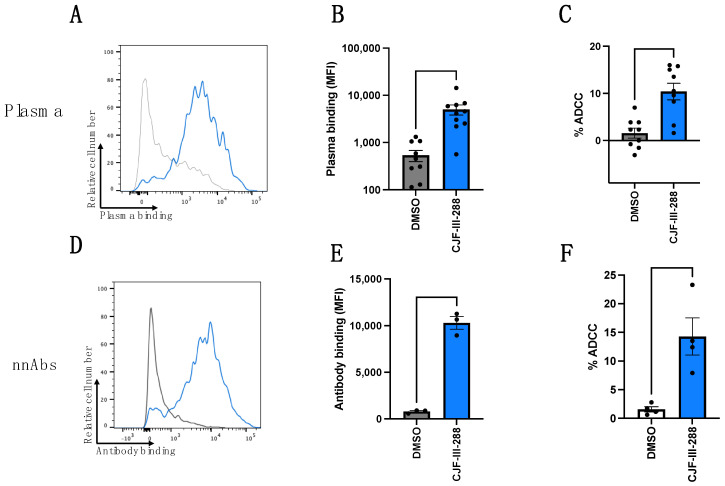
Binding and ADCC mediated by plasma from PLWH or a nnAbs cocktail. (**A**–**F**) HIV-1_CH058TF_-infected primary CD4 T cells were stained or used as target cells for ADCC with (**A**–**C**) nine PLWH plasma (dilution 1:1000) or (**D**–**F**) a total of 5 µg/mL of nnAbs antibodies in the presence of CJF-III-288 (50 µM) depicted in blue or DMSO depicted in gray 48 h post-infection. Flow cytometry was performed to detect plasma/antibody binding using an appropriated secondary antibody or to assess p24. The graph represents the mean fluorescence intensities (MFIs) of Alexa-Fluor 647 and % of ADCC obtained in at least two independent experiments, using four different cell donors. Error bars indicate means ± standard errors of the means (SEM). Statistical significance was tested using paired *t*-test based on normality.

We first evaluated the capacity of plasma from nine PLWH (Table 1) or with a cocktail of nnAbs (A32/17b/246D) to recognize infected cells in the absence or presence of the indoline CD4mc CJF-III-288 [57] (Figure 1). CD4mc are organic small-molecule HIV-1 entry inhibitors that engage the Phe43 cavity of the gp120 [62] and were shown to sensitize HIV-1-infected cells to ADCC [16] by “opening” Env, resulting in the exposure of otherwise occluded epitopes recognized by nnAbs [16,40]. The indoline CJF-III-288 CD4mc is among the most potent CD4mc that have been reported and was recently characterized in great detail [57].

Since we recently characterized the capacity of A32/17b/246D to recognize CH058TF-infected cells in combination with CD4mc CJF-III-288 at 50 µM, we kept the same virus and CD4mc concentration in the experiments outlined in this manuscript [43,63]. Briefly, primary CD4 T cells were infected with CH058TF; 48 h later, cells were stained for Env recognition by PLWH plasma or nnAbs or used to evaluate their susceptibility to ADCC. As expected, we observed a significant increase in binding and in ADCC mediated by plasma from PLWH (Figure 1A–C). While all plasma samples responded to the CD4mc, we observed an heterogeneity in this response. This is likely due to the levels of anti-cluster A, anti-CoRBS, and anti-gp41 cluster I antibodies in these plasma samples, as recently reported [63]. The cocktail of three nnAbs did not recognize infected cells nor mediate ADCC in the absence of the CD4mc (Figure 1D–F). As previously reported, an incomplete downmodulation of NTB-A and CD48 (the ligands of NTB-A and 2B4, respectively) was observed on the surface of HIV-1-infected primary CD4 T cells (Supplemental Figure S1). Importantly, CD4mc treatment did not alter cell surface levels of NTB-A and CD48 (Supplemental Figure S2).

### 3.2. NTB-A and 2B4 Contribute to ADCC-Mediated Elimination of HIV-1-Infected Cells by PLWH Plasma or by a Cocktail of nnAbs in Combination with CD4mc CJF-III-288

**Figure 2:**
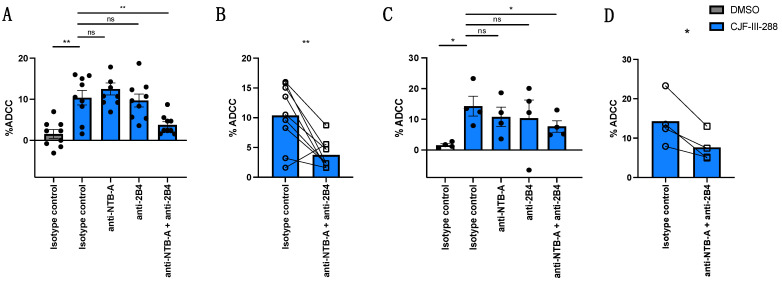
NTB-A and 2B4 modulate ADCC responses mediated by nnAbs or PLWH plasma against HIV-1-infected cells in the presence of a CD4mc. HIV-1_CH058TF_-infected primary CD4 T cells were used as target cells for ADCC with (**A**,**B**) HIV^+^ plasma (dilution 1:1000) or (**C**,**D**) a total of 5 µg/mL of nnAbs in the presence of CJF-III-288 (50 µM) depicted in blue or DMSO depicted in gray 48 h post-infection. Autologous effector cells were used as effector cells to perform ADCC killing assay. PBMCs were pre-incubated or not with anti-NTB-A and/or anti-2B4 Abs or their matched IgG isotype. The graphs represent the percentage of ADCC obtained in at least two independent experiments, using four different cell donors. Error bars indicate means ± standard errors of the means (SEM). Statistical significance was tested using paired *t*-test based on normality (*, *p* < 0.05; **, *p* < 0.01; ns, nonsignificant).

To investigate the contribution of NK cell receptors NTB-A and 2B4 in the responses observed in Figure 1, autologous PBMCs (used as effector cells; see Section 2.9 in Section 2) were pre-incubated with blocking antibodies against NTB-A or 2B4. While blocking NTB-A or 2B4 individually did not significantly affect ADCC, blocking both co-activating receptors simultaneously significantly reduced ADCC mediated by PLWH plasma (Figure 2A,B) or by the cocktail of nnAbs (Figure 2C,D). The requirement to simultaneously block both co-activating receptors to decrease ADCC might be due to the fact that NTB-A and 2B4 use the same signaling pathway to phosphorylate their immunoreceptor tyrosine-based switch motif (ITSM) and to modulate NK cell activation. This mechanism has been proposed for other NK cell activating receptors [44]. These results highlight the significant role of these co-activating receptors in NK cell activation using this strategy.

## 4. Discussion

Engaging innate immunity to purge the viral reservoir remains a timely and active area of research [2]; Fc-effector functions represent an attractive strategy to purge the viral reservoir and control viral load [3,7,64,65]. We recently developed a cocktail of nnAbs and CD4mc that efficiently eliminates HIV-1-infected primary CD4^+^ T cells and macrophages by ADCC [43]. Here, we evaluated if the ADCC response mediated by this cocktail or by plasma from PLWH in the presence of the indoline CJF-III-288 CD4mc (Figure 1) was modulated by well-known NK cell co-activating receptors. We found that blocking both co-activating receptors, NTB-A and 2B4, has a significantly effect on the elimination of infected cells by ADCC (Figure 2). These results are consistent with previous observations assessing the similar ability of both co-activating receptors to induce ADCC with the 3BNC117 bNAb [39].

**Figure 3:**
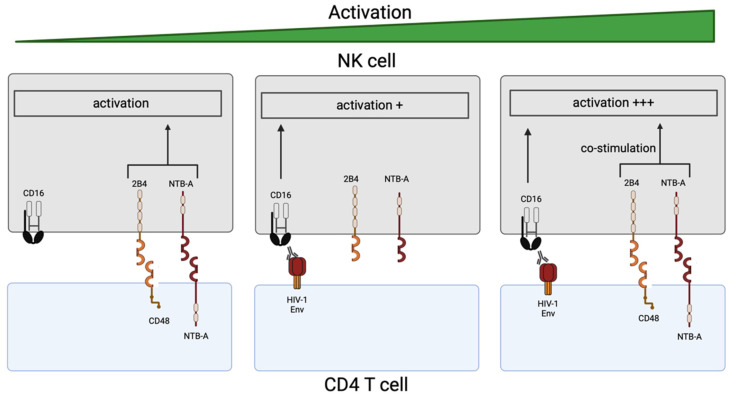
NK cell activation and ADCC in the context of HIV-1 infection. Without CD16 engagement, co-activating receptors are not sufficient to trigger NK cell degranulation (**left panel**). CD16 alone can trigger the release of lytic granules (**middle panel**) but NK cells require the engagement of co-activating receptors to sustain infected cell’s killing (**right panel**).

Our results also suggest that despite Vpu’s ability to downregulate NTB-A and CD48, the ligands for the co-activating receptors, NTB-A and 2B4, respectively, their remaining cell surface levels are sufficient to synergize with CD16. While engagement of cell surface NTB-A and CD48 with their NK cell receptors, NTB-A and 2B4 respectively, is not sufficient to trigger NK cells’ lytic activity, they do activate upon cross-linking of the CD16 receptor [36,39]. As such, the presence of Env-specific antibodies making a “bridge” between infected cells and NK cells by engaging CD16 tilts the balance toward NK cell activation (Figure 3). Our in vitro data suggest that both NTB-A and 2B4 could positively impact ADCC as a consequence of the incomplete downmodulation of their respective ligands. Future studies to evaluate the impact of both receptors on HIV-1-infected cell elimination in vivo are warranted.

Altogether, our data suggest that the complex balance of NK cell activation could be used to decrease the size of the viral reservoir by eliminating HIV-1-infected cells using CD4mc and nnAbs. While this strategy gave promising results in hu-mice by significantly decreasing the size of the reservoir in an NK-dependent manner [3], it remains to be determined if it works in additional preclinical models such as SHIV-infected non-human primates.

## Figures and Tables

**Table 1:** Cohort characteristics.

	All Samples	Chronic Infected	ART-Treated
Number of plasma samples	9	4	5
Median age	34	34	36
(IQR)	(28–56)	(28–40)	(33–56)
Sex	Male (8)	Male (4)	Male (4)
	Female (1)	Female (0)	Female (1)
Median days since	1143	1158	1018
Infection (IQR)	(792–5166)	(856–1194)	(792–5166)
Median viral load	50	35,341	50
(Copies/mL, IQR)	(40–809,600)	(29,234–809,600)	(40–50)
Median CD4 T cell count	570	416	600
(Cells/mm^3^, IQR)	(200–1149)	(200–691)	(570–1149)

## Data Availability

All data are contained within the article.

## Supplementary Materials

The following supporting information can be downloaded at: https://www.mdpi.com/article/10.3390/v16071167/s1, Figure S1. NTB-A and CD48 surface expression related to p24 expression; Figure S2. The impact of CD4mc on NTB-A and CD48 surface expression; Figure S3. Gating strategy for staining and ADCC.
