# Supplementary material for: NTB-A and 2B4 Natural Killer Cell Receptors Modulate the Capacity of a Cocktail of Non-Neutralizing Antibodies and a Small CD4-Mimetic to Eliminate HIV-1-Infected Cells by Antibody-Dependent Cellular Cytotoxicity
Source: Viruses. 2024 Jul 20;16(7):1167. doi: 10.3390/v16071167 (PMC11281563; doi:10.3390/v16071167)
Supplement: Supplementary file 1 [file viruses-16-01167-s001.zip › viruses-3079689-supplementary.pdf]

viruses

Supplemental Figure

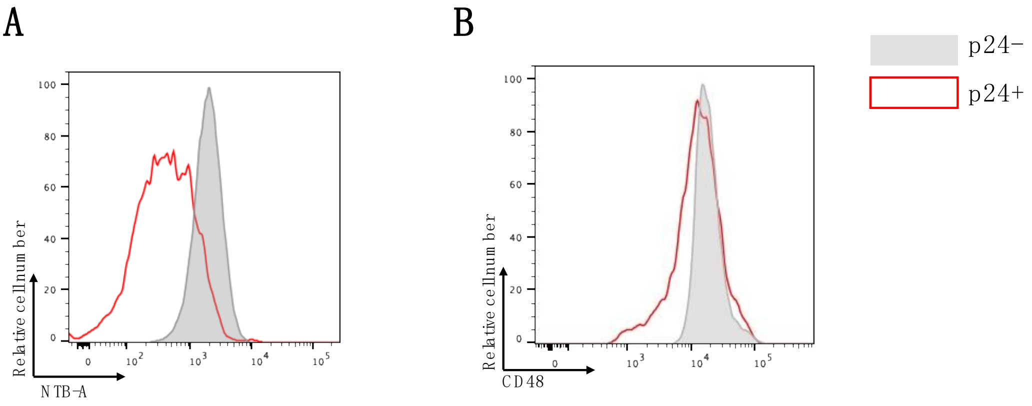

Supplemental Figure S1.NTB-A and CD48 surface expression related to p24 expression. (**A-B**) HIV-1$_{\text{CH058TF}}$-infected primary CD4 T cells were stained for **(A)** NTB-A or **(B)** CD48 48h post-infection. Flow cytometry was performed to detect antibody binding and to assess p24.

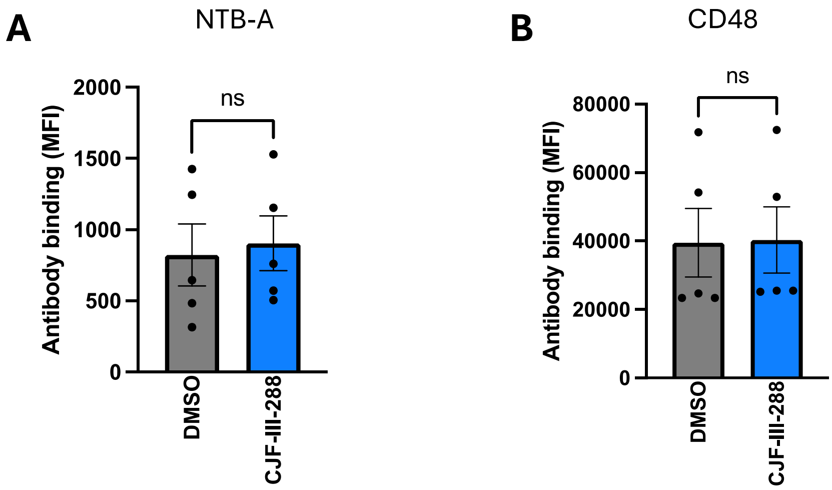

Supplemental Figure S2.NTB-A and CD48 surface expression. (**A-B**) HIV-1$_{\text{CH058TF}}$-infected primary CD4 T cells were stained for **(A)** NTB-A or **(B)** CD48 in presence of CJF-III-288 (50μM) depicted in blue or DMSO depicted in gray 48h post-infection. Flow cytometry was performed to detect antibody binding and to assess p24. The graph represents the mean fluorescence intensities (MFI) of Alexa-Fluor 647 in at least 5 independent experiments. Statistical significance was tested using (A-B) paired t-test based on normality (*, $P < 0.05$; **, $P < 0.01$; ***, $P < 0.001$; ****, $P < 0.0001$; ns, nonsignificant).

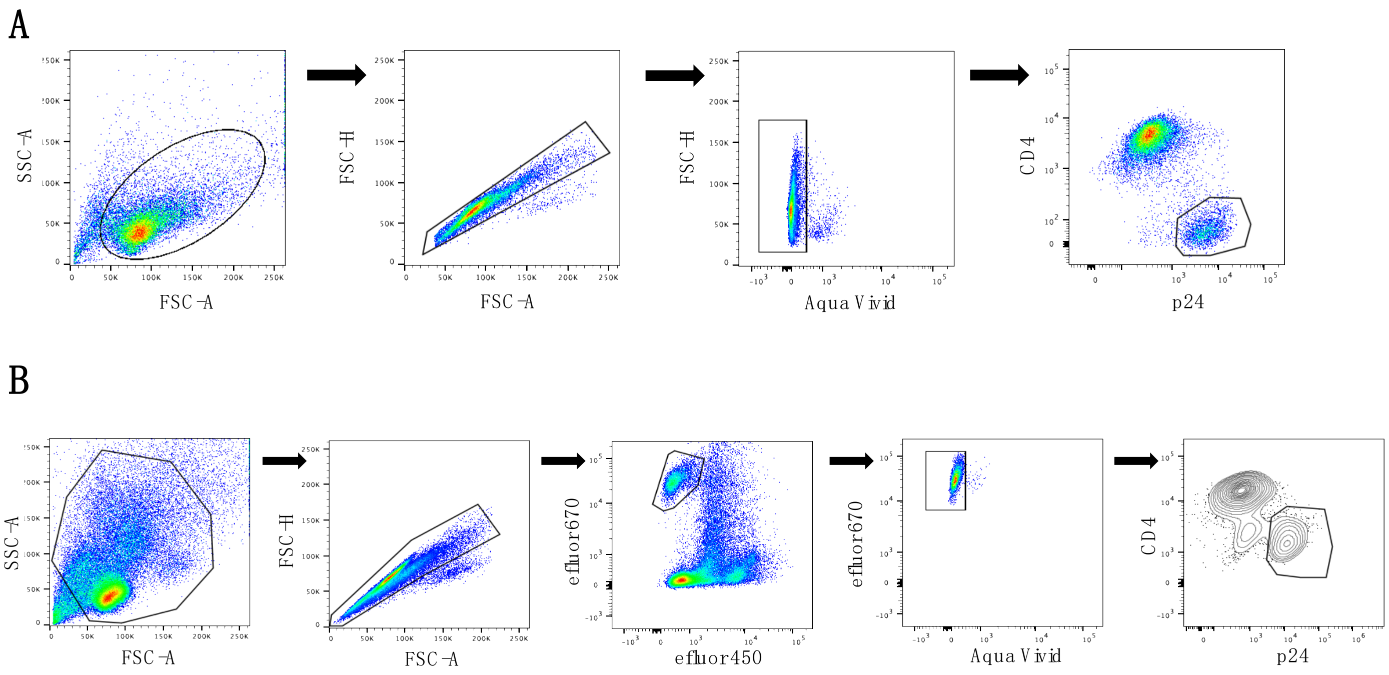

Supplemental Figure S3. Gating strategy (**A**) Staining gating strategy. Infected cells were identified by cell surface staining of CD4 and intracellular staining of p24. Mean Fluorescence Intensity (MFI) of plasma or nnAbs detected with secondary anti-human AF-647 was measured on single/live/ CD4^{low}p24^{+} cells. (**B**) Gating strategy for ADCC assay. The percentage of productively-infected cells was measured by gating on the single/eFluor670/live/CD4^{low}p24^{+} cells.
